# Karyological study of *Ololygon tripui* (Lourenço, Nascimento and Pires, 2009), (Anura, Hylidae) with comments on chromosomal traits among populations

**DOI:** 10.3897/CompCytogen.v10i4.9176

**Published:** 2016-10-10

**Authors:** Marco Antônio A. Peixoto, Marina P. C. Oliveira, Renato N. Feio, Jorge A. Dergam

**Affiliations:** 1 Laboratório de Sistemática Molecular - Beagle, Departamento de Biologia Animal, Universidade Federal de Viçosa, CEP 36570-900, Viçosa, Minas Gerais State, Brazil; 2 Museu de Zoologia João Moojen - MZUFV, Departamento de Biologia Animal, Universidade Federal de Viçosa, CEP 36570-900, Viçosa, Minas Gerais State, Brazil

**Keywords:** Cytotaxonomy, population cytogenetics, Ag-NOR, heterochromatic blocks, 18S rDNA, FISH, microsatellite DNA probes

## Abstract

To increase the number of cytogenetic characters used in *Ololygon tripui* systematics, we applied some cytogenetic techniques such as Giemsa, C- and NOR-banding, and fluorescence *in situ* hybridization (FISH) with 18S rDNA and repetitive microsatellite DNA probes to the study of four populations from Minas Gerais State (southeastern Brazil). All populations showed 2n = 24 and FN = 48, and chromosomal formula 8m + 10sm + 6st. Nucleolar organizing regions (NORs) were located on chromosome pair 6 in all populations, although in the Tripuí locality additional markings were observed on one homologue of chromosome pair 3. These patterns were partially congruent with results obtained using the 18S rDNA FISH probe. The microsatellites repetitive DNA (GA)_15_ and (CAT)_10_ probes accumulated predominantly in the terminal region of all chromosomes. Chromosome morphology and Ag-NOR were conserved among populations, a conserved pattern in *Ololygon* Fitzinger, 1843. Repetitive DNA FISH probes patterns were similar among populations, but they revealed species-specific differences when compared with other species of the genus *Ololygon*, suggesting that molecular cytogenetics are potentially more informative in karyologically conservative taxa.

fluorescence *in situ* hybridization

Nucleolar organizing regions

## Introduction

The genus *Ololygon* Fitzinger, 1843 belongs to Hylidae and currently includes 46 species (Duellman 2016, Frost 2016). In Hylidae, high level of karyotypical diversity has been reported in genera *Aplastodiscus* Lutz, 1950, *Hypsiboas* Wagler, 1830, and *Phyllomedusa* Wagler, 1830 (Carvalho et al. 2009, Ferro et al. 2012, Gruber et al. 2012, Gruber et al. 2013). On the other hand, karyotypes of *Ololygon* and *Scinax* Wagler, 1830 are highly conserved: all species show 2n = 24, and FN = 48, and morphological differences are restricted to slight variations between corresponding homeologous chromosomes (Nunes and Fagundes 2008, Cardozo et al. 2011, Nogueira et al. 2015, Peixoto et al. 2015). Within *Ololygon*, only 19 recognized species and four unnamed taxa have been karyologically studied. It’s worth mentioning that the genus *Ololygon* was resurrected from the genus *Scinax* in a recent review made by Duellman et al. 2016. Before this, the species from the genus *Ololygon* were considered as *Scinax* (Frost 2016).

In the genus *Ololygon*, the NOR is observed on chromosome pair 6, with exception of *Ololygon canastrensis* (Cardozo and Haddad, 1982), where NORs occur on chromosome pair 6 and 11 (Cardozo et al. 2011). C-banding pattern in this genus is predominantly centromeric, and some species have relatively large amount of heterochromatin (Cardozo et al. 2011, Peixoto et al. 2015). Until now, repetitive DNA probe patterns are known in only four species of this genus (reported as *Scinax* in Peixoto et al. 2015); all of them belong to the *Ololygon perpusilla* group (Peixoto 1987). In these species, repetitive DNA (CA)_15_ probe accumulated in the terminal regions of most chromosomes and showed additional markings in the centromeric region of some chromosome pairs, while the (CAT)_10_ repetitive DNA probe accumulated on the terminal or subtelomeric chromosomal regions (Peixoto et al. 2015).

Either as dispersed elements or repetitive elements organized in tandem, the repetitive DNA represents a large portion of the eukaryotic genome, and includes satellite DNA, microsatellites, minisatellites, telomeric sequences, multigene family, and transposable elements (Maxon et al. 1983, Charlesworth et al. 1994). Microsatellites sequences apparently evolve through slippage replication errors that remain unrepaired by the mechanism of DNA repair (Charlesworth et al. 1994, Jentzsch et al. 2013). Usually, these sequences accumulate close to regions characterized by low levels of recombination, such as heterochromatic regions found in terminal regions, centromeres and even in some sex chromosomes (Stephan and Walsh 2013, Cioffi et al. 2012).

Relatively a few systematic data are available for the taxonomically complex and diverse genus *Ololygon*. Thus, cytogenetic data are potentially informative for understanding the phylogenetic relationships of the species within the genus (Cardozo et al. 2011). The aim of this study was to characterize the karyotypes of four populations of *Ololygon tripui* (Lourenço et al. 2009), to explore the information potential of standard cytogenetic techniques versus FISH using18S rDNA and repetitive DNA probes. Based on results obtained, evolutionary issues relevant for this species were discussed.

## Material and methods

As a total, 32 specimens of *Ololygon tripui* were collected from four populations from the state of Minas Gerais, Brazil: Estação Ecológica do Tripuí, Ouro Preto municipality (Tripuí – Type locality), Parque Estadual Serra do Brigadeiro, Ervália municipality (PESB), RPPN Mata do Sossego, Simonésia municipality (Sossego), and Usina da Fumaça, Muriaé municipality (Fumaça) (Table 1). Proceedings were carried out according to the Animal Welfare Commission of the Universidade Federal de Viçosa and the current Brazilian laws (CONCEA 1153/95). All vouchers were housed in the herpetological collection of the Museu de Zoologia João Moojen at the Universidade Federal de Viçosa (MZUFV), Viçosa municipality, in Minas Gerais State, Brazil (Table 1).

Mitotic chromosomes were obtained from gut epithelial cells according to Schmid (1978). Each specimen was injected intraperitoneally with 0.1% solution of colchicine (0.1 ml per 10 g of body weight) for 4 hours before euthanasia (carried out with 5% lidocaine). Best metaphases were photographed in digital Olympus BX53 light microscope with a DP73 Olympus camera. Chromosome pairing and measurements were performed using Image Pro Plus® (IPP Version 4.5) to determine the modal value (2n) and the FN for each population. Homologs were paired and grouped according to the centromere position, in decreasing size order. Approximately 20 metaphase spreads were analyzed per specimen, to determine the diploid chromosome number and karyotypic structure. The chromosomes were classified according to their centromeric indices in metacentric (m), submetacentric (sm) and subtelocentric (st) following Green and Sessions (1991).

**Table 1.** Sample sizes (N) per gender, sample locality and voucher identification of *Ololygon tripui* populations.

Population	Sample locality	N	Gender	Voucher (MZUFV)
PESB	Parque Estadual Serra do Brigadeiro, Ervália – MG (20°51'52"S, 042°31'17"W)	9	Male	9865, 9870, 9871, 9872, 10294, 10299-301, 11421
		3	Female	11511, 12103-104
Sossego	RPPN Mata do Sossego, Simonésia – MG (20°04'22"S, 042°04'12"W)	6	Male	11441-443, 12571, 12575-576
		2	Female	12573, 12577
Fumaça	Usina da Fumaça, Muriaé – MG (21°00'58"S, 042°26'36"W)	6	Male	11444-449
		3	Female	11438-440
Tripuí	Estação Ecológica do Tripuí, Ouro Preto – MG (20°23'22"S, 043°32'20"W)	3	Male	12447-449

To identify heterochromatic regions, the C-banding technique followed Schmid et al. (1979). Active NORs in the preceding interphase were identified using silver nitrate precipitation (Ag-NOR) following Howell and Black (1980). The technique of FISH was performed according to Pinkel et al. (1986). The 18S rDNA probe was obtained from *Ololygon tripui* via PCR and it was labeled by nick-translation with biotin-14-dATP. Signal detection and amplification were performed using isothiocyanate probe, fluorescein-conjugated avidin and anti-avidin-biotin. The (GA)_15_ and (CAT)_10_ repetitive DNA probes were thynilated with cy3 at the 5’ position (Sigma-Aldrich). FISH images were captured in a BX53 Olympus microscope with a XM10 camera.

## Results

**Figure 1.**
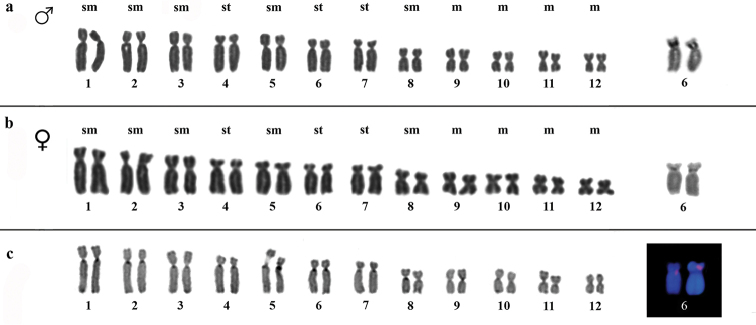
**a–c**. Karyotype of *Ololygon tripui* from the Fumaça locality. **a** Giemsa and Ag-NOR staining of male chromosomes **b** Giemsa and Ag-NOR staining of female chromosomes **c** C-banding and 18S rDNA markers on chromosome pair number 6. Bar = 10 µm.

**Figure 2.**
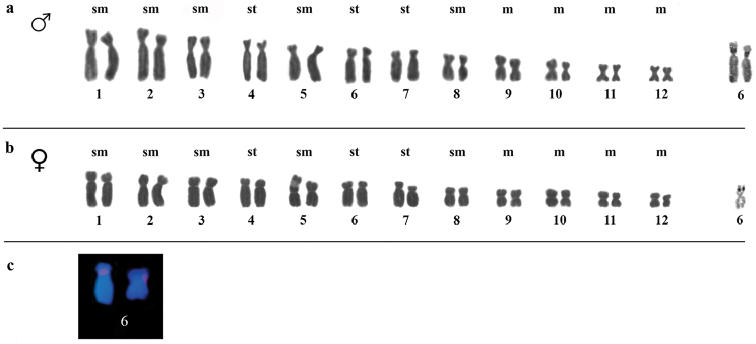
**a–c**. Karyotype of *Ololygon tripui* from PESB locality. **a** Giemsa and Ag-NOR staining of male chromosomes **b** Giemsa and Ag-NOR staining of female chromosomes **c** 18S rDNA sites on chromosome pair number 6. Bar = 10 µm.

**Figure 3.**
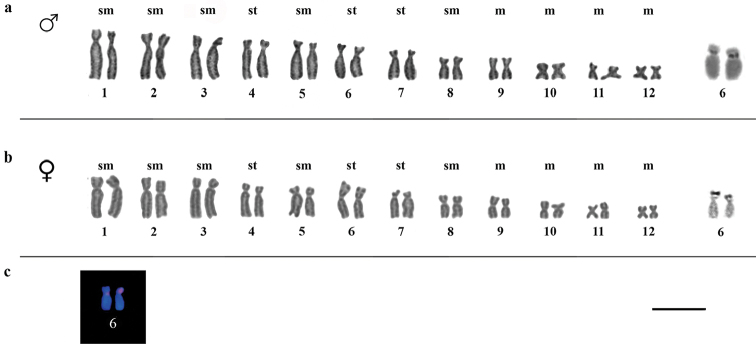
**a–c**. Karyotype of *Ololygon tripui* from the Sossego locality. **a** Giemsa and Ag-NOR staining of male chromosomes **b** Giemsa and Ag-NOR staining of female chromosomes **c** 18S rDNA markers on chromosome pair number 6. Bar = 10 µm.

**Figure 4.**
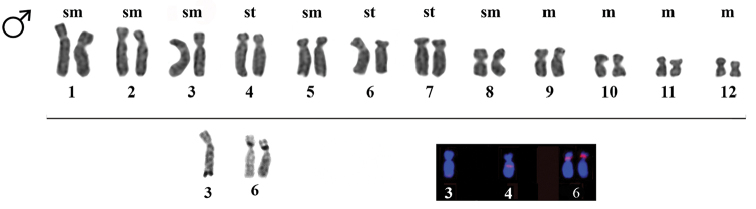
Karyotype of *Ololygon tripui* from the Tripuí locality. Giemsa, Ag-NOR staining, and 18S rDNA markers on multiple chromosomes of a male specimen. Bar = 10 µm.

All the individuals analyzed had a diploid complement of 24 chromosomes. All chromosomes were biarmed and their FN was 48 (Suppl. material 1 and Table 2). The chromosomal formulae of the *Ololygon tripui* was 8m + 10sm + 6st, whereas chromosome pair 1, 2, 3, 5, and 8 were sm; 4, 6, and 7 were st, and 9–12 were m in all populations (Figs 1–4).

**Table 2.** Comparative morphology and measurements of chromosome pairs in *Ololygon tripui* populations. m = metacentric, sm = submetacentric, st = subtelocentric, CR = centromeric ratio, CT = chromosome type.

Population	Gender	Chromosome pairs												
			1	2	3	4	5	6	7	8	9	10	11	12
PESB	Female	CR	2.41	2.53	2	3.38	2.46	3.25	4.55	2.4	1.25	1.11	1.23	1.19
		CT	sm	sm	sm	st	sm	st	st	sm	m	m	m	m
	Male	CR	2.11	2.32	2.3	3.57	1.99	3.4	3.19	2.31	1.1	1.1	1.1	1.17
		CT	sm	sm	sm	st	sm	st	st	sm	m	m	m	m
Sossego	Female	CR	2.38	2.28	2.41	3.91	2.04	3.96	3.63	2.17	1.49	1.35	1.15	1.26
		CT	sm	sm	sm	st	sm	st	st	sm	m	m	m	m
	Male	CR	2.17	2.18	2.09	3.49	2.1	3.45	3.53	2.05	1.23	1.32	1.19	1.14
		CT	sm	sm	sm	st	sm	st	st	sm	m	m	m	m
Fumaça	Female	CR	2.58	2.62	1.97	3.68	2.56	3.66	3.53	2.13	1.45	1.33	1.28	1.16
		CT	sm	sm	sm	st	sm	st	st	sm	m	m	m	m
	Male	CR	2.3	2.42	1.8	3.46	2.06	3.57	3.95	2.31	1.31	1.33	1.2	1.28
		CT	sm	sm	sm	st	sm	st	st	sm	m	m	m	m
Tripuí	Male	CR	2.39	2.44	2.51	3.76	2.31	3.67	3.67	1.81	1.35	1.19	1.15	1.14
		CT	sm	sm	sm	st	sm	st	st	sm	m	m	m	m

Heterochromatic blocks were detected in the centromeric and pericentromeric regions of chromosomes, only in the specimens from the Fumaça locality. Heterochromatic patterns were similar in both sexes (Fig. 1). The Ag-NOR region was pericentromeric and located on the short arm of chromosome pair 6. However, in the PESB specimens, the Ag-NOR was restricted to only one chromosome in females. In addition to marking the chromosome pair 6, males from the Tripuí population showed an additional Ag-NOR in the subtelomeric region in the long arm on one homolog of chromosome pair 3 (Fig. 4). The presence of a secondary constriction associated with Ag-NOR was also detected in the chromosome pair 6, and sometimes it was evident in only one of the homologs.

**Figure 5.**
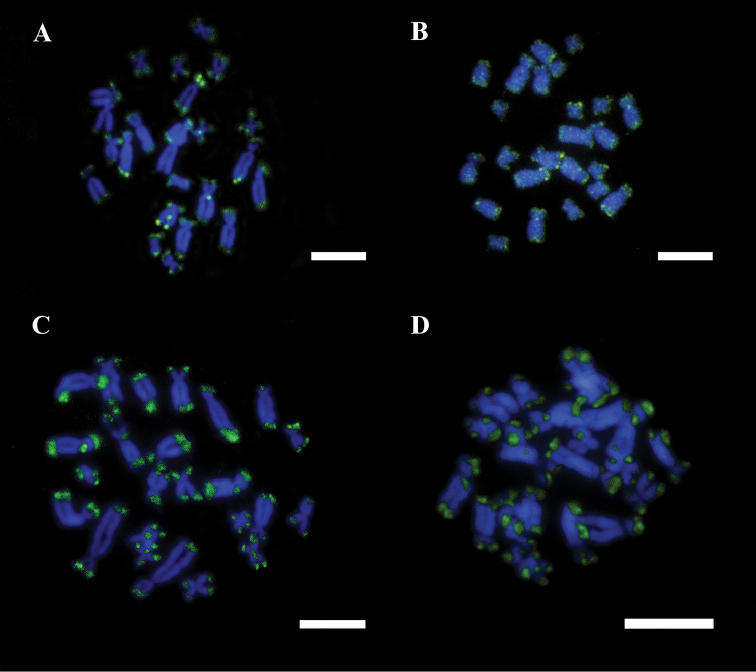
**a–d.** Male mitotic chromosomes of *Ololygon tripui* from different populations, labeled with the (GA)_10_ repetitive DNA probe. **a** Fumaça **b** PESB **c** Sossego **d** Tripuí. Bar = 10 µm.

**Figure 6.**
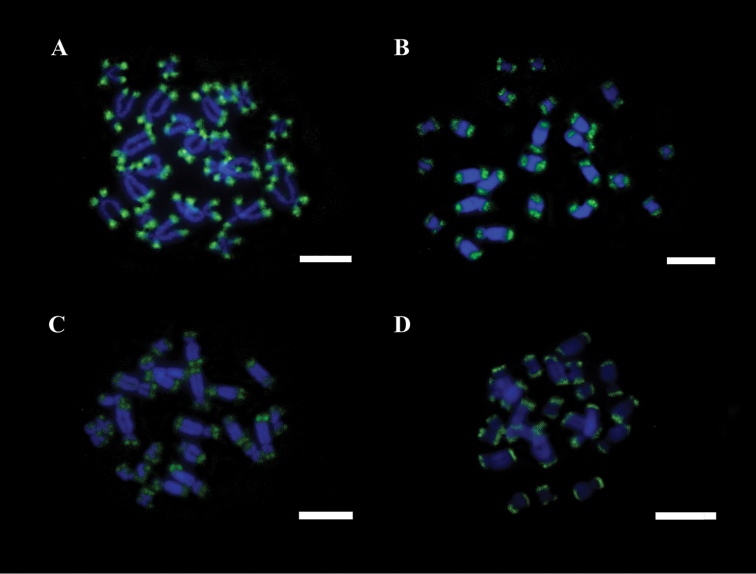
**a–d.** Male mitotic chromosome of *Ololygon tripui* from different populations, labeled with the (CAT)_15_ repetitive DNA probe. **a** Fumaça **b** PESB **c** Sossego **d** Tripuí. Bar = 10 µm.

FISH showed 18S rDNA sites in chromosome pair 6 in males and females from the Fumaça, PESB, and Sossego populations. Multiple tags for 18S rDNA FISH were also observed in a male of the Tripuí population, with markings restricted to one homolog in chromosome pair 3 and 4, and on both homologs of chromosome pair 6. The microsatellites repetitive DNA probe (GA)_15_ accumulated in the terminal region of all chromosomes (Fig. 5), whereas the (CAT)_10_ probe showed conspicuous accumulation in the terminal regions of all chromosomes, with additional markings in the centromeric regions of some chromosomes (Fig. 6).

## Discussion

The diploid number of 2n = 24 and FN = 48 observed in *Ololygon tripui* was similar to those reported for all studied species of the genus and considered as a highly conserved character, which is shared with other genera within Hylidae (i.e. *Scinax*, *Xenohyla* Izecksohn, 1998, and *Lysapsus* Cope, 1862) (Suárez et al. 2013). However, our results suggest that other characters associated with *Ololygon*, may represent putative synapomorphy of species within the genus *Ololygon*: i) *Ololygon tripui* showed the chromosome pair 1 and 2 submetacentric-like and the chromosome pair 6 subtelocentric-like as well as all karyologically studied species of the genus. In *Ololygon belloni* Faivovich, Gasparini and Haddad, 2010, the chromosome pair 1 is metacentric, which is interpreted as a possible autapomorphy of this species (Peixoto et al. 2015); ii) Ag-NOR cistrons were identified on the chromosome pair 6 in all populations of *Ololygon tripui*. These traits place the species of *Ololygon* as a chromosomally well-differentiated taxon from the species of *Scinax*. In the latter genus, the chromosome pair 1 and 2 are metacentric-like, the chromosome pair 6 is submetacentric-like; the Ag-NOR cistrons, as in most other hylids, usually occur on the chromosome pair 11. The only exception is *Scinax alter* (Lutz, 1973), which has terminal NORs on chromosome pair 3 (Nunes and Fagundes 2008, Cardozo et al. 2011, Peixoto et al. 2015). The genus *Ololygon* was recently resurrected from the genus *Scinax* (Duellman et al. 2016), based on molecular data. Our study demonstrates that chromosomal characters are also congruent with this taxonomical decision.

The polymorphic presence of more than one pair of Ag-NOR cistrons in the Tripuí population of *Ololygon tripui* have also been reported in other species of this genus (Cardozo et al. 2011). For instance, in *Ololygon rizibilis* (Bokermann, 1964) Ag-NORs are present on chromosome pair 5 and 6 from two different populations, whereas in *Ololygon canastrensis*, Ag-NORs occurs on chromosome pair 6 and 11 (Cardozo et al. 2011). In *Ololygon tripui*, the secondary constriction, associated with NORs, was found in the interstitial region close to the centromeric region of chromosome pair number 6, but sometimes it was restricted to one member of this chromosome pair. In anurans, the correlation of Ag-NORs with secondary constriction is commonly observed (Barth et al. 2009) and characterizes *Ololygon* (Cardozo et al. 2011).

Markings obtained with 18S rDNA FISH revealed some chromosomal aspects that were not evident using the Ag-NOR protocol. Although high correlation was observed in the Fumaça and Sossego populations between the FISH and NOR banding patterns, in the PESB population this correlation was partial: only one homolog showed Ag-NOR banding, whereas both homologs bore 18S rDNA sites. Notably, in the Tripuí population, 18S rDNA sites were detected in one homolog of chromosome pair 3 and 4 and in both homologs of chromosome pair 6; Ag-NOR-banding was evident on one homolog of chromosome pair 3, and on the chromosome pair 6. Unlike Ag-NORs, FISH exposure of rDNA regions occurs regardless of previous interphase activation of this region, allowing to detect actual levels of polymorphisms between populations or species (Zurita et al. 1998). In fact, the results of the 18S rDNA FISH are more representative in PESB and Tripuí populations.

Partial correlation between results obtained by Ag-NOR and FISH techniques can be explained by the action of different mechanisms, such as: i) a limitation of the technique in detecting a rDNA sequences present in low copy number; ii) the mobility of rDNA sequences spread by transposable elements; iii) a real polymorphic condition; iv) the occurrence of chromosome rearrangements such as translocations; v) the physiological amplification of rDNA cistrons (Macgregor and Kezer 1973, Schubert and Wobus 1985, King et al. 1990).

Chromosome mapping using microsatellite sequences in hylids is scarce, however available data suggest that this kind of sequences accumulate in the vicinity of centromeres and telomeres (Peixoto et al. 2015). The accumulation of trinucleotide DNA probe (CAT)_10_ in the terminal regions plus additional markings in the centromere region of some chromosomes of *Ololygon tripui* populations, was similar to the pattern observed in bromeligenous species of *Ololygon* (*Ololygon* sp., *Ololygon arduoa* Peixoto, 2002, *Ololygon belloni*, and *Ololygon cosenzai* Lacerda, Peixoto and Feio, 2012), where this probe are restricted to the terminal or subtelomeric regions of chromosomes (Peixoto et al. 2015). The dinucleotide (GA)_15_ probe showed a conserved pattern in the populations of the *Ololygon tripui* reported in this study. The (CA)_10_ also showed accumulation in terminal regions of chromosomes, plus additional markings on centromeric regions of some chromosomes of the other *Ololygon* species (Peixoto et al. 2015).

Our results indicate that despite *Ololygon tripui* populations were conservative in most of their cytogenetic characters such as diploid and fundamental number, Ag-NOR, repetitive DNA, and 18S rDNA patterns in relation to *Ololygon* sp., *Ololygon arduoa*, *Ololygon belloni*, and *Ololygon cosenzai*, the former species differs in its chromosome morphology and in the repetitive DNA pattern, that probably occurs due to independent evolution of the species, thus corroborating its taxonomic status.

We concluded that although repetitive DNA patterns of variation are largely unknown in anurans, the cytogenetic mapping of different repetitive DNA sequences provided reliable chromosomal markers, revealing species-specific differences, when compared with other species of the genus. Our study showed initial insights on the use of repetitive DNA probes to discuss evolutionary issues.

## Supplementary materials

Supplementary material 1 Data on chromosomal and fundamental number, chromosomal formulae, C and NOR banding, 18S, and sample locality to all species from the genus *Ololygon* with some karyotypic study Data type: molecular data This dataset is made available under the Open Database License (http://opendatacommons.org/licenses/odbl/1.0/). The Open Database License (ODbL) is a license agreement intended to allow users to freely share, modify, and use this Dataset while maintaining this same freedom for others, provided that the original source and author(s) are credited.Marco Antônio A. Peixoto, Marina P. C. Oliveira, Renato N. Feio, Jorge A. Dergam
